# Comparison of quality of life and arm complaints after axillary lymph node dissection *vs* sentinel lymph node biopsy in breast cancer patients

**DOI:** 10.1038/sj.bjc.6601150

**Published:** 2003-08-12

**Authors:** F Peintinger, R Reitsamer, H Stranzl, G Ralph

**Affiliations:** 1 Gynecological Department, General Hospital Bruck/Leoben, Vordernbergerstrasse 42, 8700 Leoben, Austria; 2 Department for Breast Diseases, General Hospital Salzburg, Muellner Hauptstrasse 42, 5020 Salzburg, Austria; 3 Department for Radiotherapy, University Medical School of Graz, Auenbruggerplatz 32, 8036 Graz, Austria

**Keywords:** quality of life, sentinel lymph node biopsy, breast cancer, morbidity

## Abstract

The sentinel lymph node biopsy (SLNB) represents a minimal invasive surgical method for axillary staging in patients with primary breast cancer. In a prospective study, evaluation of quality of life (QOL) and arm morbidity was performed before surgery on a total of 56 breast cancer patients. The EORTC QLQ-C30 and EORTC QLQ-BR23 questionnaires were used for QOL assessment. Assessment of pain was additionally observed using the McGill Pain Questionnaire. Arm mobility was observed by goniometric measurement of arm movement. Data were collected before surgery (t1), 1 week after discharge (t2) and 9–12 months after surgery (t3).

The type of axillary surgery does not seem to affect global QOL at a short-time follow-up, but patients recover sooner after SLNB. Body image and sexual functioning remain stable in both types of axillary surgery. Arm/shoulder pain was reported in 36% of patients after SLNB in comparison to 68% receiving axillary lymph node dissection (ALND), and ‘numbness’ was reported only in 4% of patients in the SLNB group *vs* 19.3% after ALND. Abduction, flexion and horizontal adduction of the affected arm show significant impairment after ALND. Breast cancer patients should be counselled about the benefits of SLNB over ALND concerning QOL and postsurgery side effects in a short-term follow-up.

Axillary lymph node dissection (ALND) in breast cancer patients still represents the routine surgical method for axillary staging. Although the axillary node status is the most important prognostic factor for recurrence and survival (Fisher *et al*, 1984; Carter *et al*, 1989) and information obtained by axillary dissection is useful for planning adjuvant treatment, it is associated with substantial morbidity (Kissin *et al*, 1986; Ivens *et al*, 1992; Keramopoulos *et al*, 1993; Hack *et al*, 1999; Kakuda *et al*, 1999) and psychological distress (Maunsell *et al*, 1993; Tobin *et al*, 1993; Shimozuma *et al*, 1999). Hack *et al*, showed arm/shoulder pain, weakness or numbness in 72% and impaired range of motion in 73% of breast cancer patients after ALND, whereas high levels of quality of life (QOL) were reported. Moderate to severe pain was reported between 20, 23 and 32% (Van Dam *et al*, 1993; Kuehn *et al*, 2000; Ververs *et al*, 2001) and was not significantly related to time since surgery. Other reports suggest that arm problems after ALND are associated with a negative effect on the overall QOL of breast cancer patients (Maunsell *et al*, 1993; Kuehn *et al*, 2000). As a result of the need to reduce axillary morbidity, many investigations have been performed on sentinel lymph node biopsy (SLNB), an alternative procedure. Using vital dye and/or radiocolloid, the sentinel node/s as the first lymph node to receive lymphatic drainage from the primary tumour can be identified by a minimal invasive surgical technique. Recently, published data showed no sensory morbidity after SLNB (Giuliano *et al*, 2000) at a median follow-up of 39 months. Schrenk *et al* (2000) reported less postoperative arm pain, numbness and arm motion restriction after SLNB at a follow-up period of 15.4 months. The evaluation of morbidity after ALND *vs* SLNB is under investigation in ongoing randomised trials as the NSABP B-32 and the ALMANAC trial. The evaluation of QOL issues such as treatment side effects, patients satisfaction and symptom management are substantial parameters in decision making regarding surgical interventions. However, at this time little is known about the impact of SLNB on QOL in breast cancer patients.

The major objectives of this study are (1) to evaluate QOL differences in a short-term follow-up after two surgical procedures (ALND and SLNB) in breast cancer patients receiving breast-conserving treatment; (2) to determine the impact of SLNB on global QOL of breast cancer patients and (3) to compare morbidity end points (arm/shoulder mobility, pain, sensory morbidity) during different clinical phases.

## MATERIALS AND METHODS

### Selection of patients

In a prospective, longitudinal study between September 2000 and March 2002, we included 56 consecutive patients with newly diagnosed primary breast cancer. Study eligibility criteria included the following: (1) breast cancer stage I or II, (2) breast-conserving surgery in all patients, (3) patients' age between 18 and 80 years, (4) no severe physical and mental comorbidity, (5) performance status 0 and (6) informed consent.

### Procedures

A total of 56 patients with invasive breast cancer received the sentinel node biopsy. In all, 25 patients receiving the SLNB only (Group I) were compared with 31 patients who underwent the standard level I and II ALND (Group II) when intraoperative frozen section showed metastatic disease. Before the study was started, a surgical protocol was implemented in order to minimise differences in technique. Similar incisions, similar anatomic dissections and similar drainage catheters were used. All patients received breast-conserving surgery. Our technique of SLNB has been described previously (Reitsamer *et al*, 2002). Briefly, SLNB was performed by the combined method using peritumoral injection of technetium-99m-labelled albumin (Nanocoll®, Sorin Biomedica, Saluggia, Italy) and subareolar subcutaneous injection of blue dye (Patent Blue V®, Laboratoire Guerbet, Aulnay-sous-Bois, France). Technetium-99m was injected 16–18 h before surgery and blue dye was injected 5 min prior to incision to identify the SLN. Hot and blue nodes were removed and frozen section was performed immediately. If SLN/s were negative in frozen section, patients had no further ALND. All patients received whole-breast irradiation after surgery. No radiotherapy to the axilla was performed. Adjuvant chemotherapy was administered before radiotherapy when indicated. Adjuvant endocrine treatment was initiated after surgery. The decision to use adjuvant chemotherapy or hormone therapy was mainly based on prognostic factors from the primary breast tumour such as tumour size, hormone receptor status and/or HER-2/neu status. Additionally, node-positive patients received adjuvant hormone therapy by participating in the national hormone treatment trial.

### Assessments

#### Frequency

Data were collected at three time points: before surgery (t1), 1 week after discharge (t2), and 9–12 months after surgery (t3).

#### EORTC QLQ-C30

The European Organization for Research and Treatment of Cancer Quality of Life Questionnaire QLQ-C30, version 3.0, a cancer-specific questionnaire, is composed of five functional scales (physical, role, emotional, cognitive, social), the global health status and nine symptom scales (fatigue, nausea and vomiting, pain, dyspnoea, insomnia, appetite loss, constipation, diarrhoea, financial difficulties). The global health status correlates significantly with all the functional and symptom scales (Aaronson *et al*, 1993). For the functional and global QOL scales a higher score indicates a better level of functioning. All patients answered this questionnaire before surgery (t1), 1 week after discharge (t2) and 9–12 (t3) months after surgery.

#### EORTC QLQ-BR23

The European Organization for Research and Treatment of Cancer Quality of Life Questionnaire QLQ-BR23, the breast cancer module, incorporates four symptom scales (systemic therapy side effects, breast symptoms, arm symptoms, upset by hair loss) and four functional scales (body image, sexual functioning, sexual enjoyment, future perspective). All scores obtained from scales and single items range from 0 to 100. A higher score indicates a better level of functioning. All patients answered this questionnaire before surgery (t1), and 9–12 months after surgery (t3).

#### Range of arm/shoulder motion

All patients underwent goniometric measurement of the affected arm by a physiotherapist at every time point. Measurements of the following arm movements were obtained: shoulder flexion, shoulder extension, shoulder abduction, horizontal abduction and horizontal adduction.

#### McGill Pain Questionnaire

German version (Melzack, 1975; Stein and Mendl, 1988). This questionnaire is composed of sensory, affective, evaluative word descriptors in the form of 78 words grouped into 20 subclasses used by patients to specify subjective pain experience and of a visual analogue pain scale for measurement of pain intensity. The questionnaire provides information about the site of pain and the relative effects of a given manipulation on several dimensions of pain. All patients completed the questionnaire at every time point.

#### Karnofsky performance status scale (KPS)

The KPS scale consists of 11 components describing patients' mobility and ability to maintain employment, live at home and care for oneself. The scores used by clinicians range from 0 (worst physical condition) to 100 (best physical status).

### Statistical analysis

Statistical methodology was used in accordance with *The EORTC QLQ-C30 Scoring Manual* (Fayers *et al*, 1999). In order to compare both types of surgery (ALND *vs* SLNB) nonparametric independent two-sided tests were applied (Wald–Wolfowitz test, Kolmogorov–Smirnov test, Mann–Whitney *U*-test) to all variables tested. Differences of the proportions of patients reporting pain after ALND over time were analysed using the Cochran's *Q* test. The same test was applied for analysis of the SLNB group. A *P*-value less than 5% was considered as significant.

## RESULTS

In all, 56 breast cancer patients participated in this study. Patients' clinical and demographic characteristics are summarized in Table 1

**Table 1:** Patient characteristics

	**Total sample(*n*=56)**	**ALND(*n*=31)**	**SLNB(*n*=25)**	***P*-value**
*Age (years)*				
Mean	60.1	57.7	61.4	0.27
*Site of axillary surgery* (%)				
Right axilla	51.7	54.8	48.0	0.62
Left axilla	48.3	45.2	52.0	0.6
*Cancer type* (%)				
Invasive ductal	62.5	64.5	56.0	0.52
Invasive lobular	14.3	6.5	24.0	0.07
Invasive ductal and intraductal	23.2	29.0	20.0	0.44
*Tumour size* (%)				
pT1	71.4	74.2	68.0	0.62
pT2	28.6	25.8	32.0	0.62
*Adjuvant treatment* (%)				
HT and RT	67.9	67.7	68.0	0.62
CT and RT	23.2	29.0	20.0	0.44
RT alone	8.9	3.3	12.0	0.22
*Menopausal status* (%)				
Premenopausal	28.6	35.5	20.0	0.20
Postmenopausal	71.4	64.4	80.0	0.20
*Civil status* (%)				
Married/partnership	53.6	58.1	48.0	0.45
Single/widowed/divorced	30.4	35.5	24.0	0.36
Unknown	16.0	6.4	28.0	0.03
*Education* (%)				
Primary	44.6	51.6	40.0	0.39
Secondary/professional	25.0	25.8	24.0	0.86
Higher	14.3	16.1	12.0	0.66
Unknown	16.1	6.5	24.0	0.06

HT=hormone therapy; RT=radiotherapy; CT=chemotherapy.

. The mean number of lymph nodes dissected was 2.2 in group I and 15.0 in group II. The percentage of postmenopausal patients was 71.4%. Preoperative arm symptoms, the Karnofsky performance status and QOL levels were comparable between both groups. Table 2

**Table 2:** Means (s.d.) of the EORTC QLQ-C30 scale scores

	**ALND**	**SLNB**	***P*-value**
*Before surgery (t1)*			
Global QOL	63.8 (24.9)	55.9 (25.9)	0.87
Physical functioning	87.7 (19.2)	90.8 (17.9)	0.69
Role functioning	88.0 (26.1)	90.9 (17.7)	0.69
Emotional functioning	65.3 (21.6)	53.8 (31.1)	0.05
Cognitive functioning	80.6 (21.9)	84.0 (23.8)	0.41
Social functioning	85.9 (23.6)	84.8 (22.3)	0.75
Pain	14.1 (23.2)	18.7 (27.0)	0.90
			
*After discharge (t2)*			
Global QOL	57.8 (20.5)	68.5 (17.0)	0.58
Physical functioning	82.3 (15.4)	86.7 (14.9)	0.37
Role functioning	60.2 (28.0)	70.4 (30.9)	0.37
Emotional functioning	75.6 (21.3)	70.7 (27.3)	0.07
Cognitive functioning	86.1 (22.3)	90.7 (14.7)	0.65
Social functioning	78.7 (28.4)	83.4 (22.0)	0.65
Pain	34.3 (29.2)	16.7 (20.4)	<0.05^a^
			
*9–12 months after surgery (t3)*			
Global QOL	72.1 (22.7)	70.2 (20.3)	0.45
Physical functioning	85.9 (21.4)	87.2 (18.2)	0.34
Role functioning	74.1 (27.6)	78.3 (26.8)	0.63
Emotional functioning	68.9 (19.8)	70.5 (25.4)	0.63
Cognitive functioning	77.6 (25.3)	82.6 (24.8)	0.21
Social functioning	86.2 (23.6)	89.8 (19.9)	0.50
Pain	21.3 (25.9)	18.8 (24.8)	0.29

a Statistically significant.

provides QOL levels (EORTC QLQ-C30) at all time points of assessment. The mean scores at baseline assessment (t1) showed that patients' global QOL and emotional functioning were more affected in both groups than physical functioning, role functioning, cognitive functioning and social functioning. However, significant improvement of global QOL (*P*=0.002) occurs at t2 only in patients after SLNB (Figure 1

**Figure 1:**
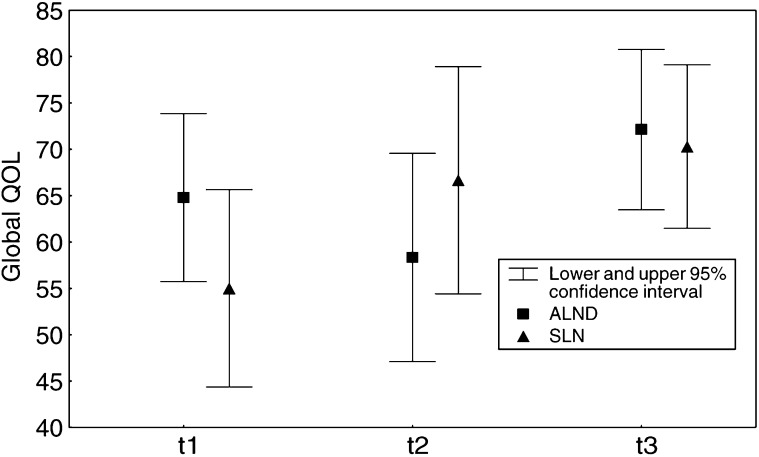
Comparison of global QOL (means) after ANLD *vs* SLNB over time.

). Analysis of means of symptom scales shows significant higher levels of pain at t2 in patients after ALND (*P*=0.03) (Figure 2

**Figure 2:**
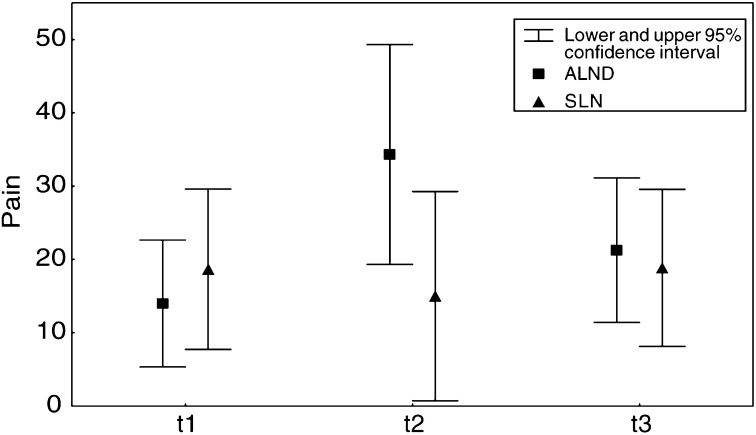
Comparison of pain (means) after ALND *vs* SLNB over time.

). Comparison of QOL dimensions assessed by the EORTC QLQ-C30 and QLQ-BR23 (Tables 2 and 3

**Table 3:** Means (s.d.) of the EORTC QLQ-BR23 scale scores and Karnofsky performance status

	**ALND**	**SLNB**	***P*-value**
*Before surgery (t1)*			
Body image	89.9 (18.3)	83.2 (23.7)	0.39
Sexual functioning	30.9 (31.9)	28.7 (27.7)	0.69
Sexual enjoyment	61.5 (38.1)	52.3 (32.5)	0.95
Future perspective	38.8 (33.9)	31.8 (39.5)	0.56
Arm symptoms	8.3 (14.4)	19.5 (25.2)	0.07
			
KPS	98.2 (4.6)	99.1 (2.8)	0.10
			
*9–12 months after surgery (t3)*			
Body image	87.9 (15.8)	92.0 (13.6)	0.45
Sexual functioning	33.4 (33.4)	35.1 (24.8)	0.45
Sexual enjoyment	63.9 (26.4)	66.7 (23.5)	0.20
Future perspective	56.7 (32.9)	54.5 (34.9)	0.18
Arm symptoms	21.2 (22.8)	14.0 (18.4)	0.26
			
KPS	96.1 (18.1)	99.5 (2.0)	0.21

) shows no statistically significant differences among patients in both groups before surgery (t1). At t3, global QOL improved in both groups, but there were no statistically significant differences in any dimension of QOL. Karnofsky performance scores at baseline were high in both groups and showed no significant changes over time (Table 3).

Analysis of arm/shoulder mobility assessment data showed significant impairment of abduction and flexion in the operated arm at the time points t2, t3 and of horizontal adduction at the time point t3 in group II (Table 4

**Table 4:** Means (s.d.) arm/shoulder motion, goniometric measurement

	**ALND**	**SLNB**	***P*-value**
*Before surgery (t1)*			
Abduction	153.7 (19.3)	160.4 (10.9)	0.75
Flexion	152.1 (17.9)	153.6 (13.6)	0.58
Extension	50.7 (8.4)	49.3 (5.8)	0.55
Horizontal abduction	108.6 (12.8)	108.1 (12.4)	0.55
Horizontal adduction	35.1 (14.0)	32.2 (8.2)	0.98

*After discharge (t2)*			
Abduction	128.3 (24.9)	152.3 (13.7)	0.013*
Flexion	134.8 (21.9)	150.6 (16.1)	0.04*
Extension	48.6 (11.5)	51.7 (5.0)	0.58
Horizontal abduction	106.1 (15.8)	108.4 (13.2)	0.72
Horizontal adduction	29.5 (14.4)	29.4 (11.3)	0.72
			
*9–12 months after surgery (t3)*			
Abduction	143.8 (22.8)	158.9 (13.9)	0.007*
Flexion	146.0 (15.9)	154.6 (15.0)	0.03*
Extension	47.1 (11.2)	52.2 (27.1)	0.39
Horizontal abduction	101.1 (15.9)	106.5 (21.3)	0.76
Horizontal adduction	34.5 (14.1)	35.6 (19.1)	0.011*

* *P*<0.05.

).

Analysis of data assessed by the McGill Pain Questionnaire showed significantly more sensory problems of the affected arm in group II at t3 when the number of words chosen (NWC) was compared with those of group I. Severity of pain measured by the visual analogue scale showed that women in group II reported significantly greater pain than in group I at t3 (Table 5

**Table 5:** Means (s.d.) for pain

	**ALND**	**SLNB**	***P*-value**
*After discharge (t2)*			
Sensory (NWC)	2.29 (2.67)	0.88 (1.45)	0.552
Visual analogue scale	1.45 (1.36)	0.68 (1.03)	0.823
			
*9–12 months after surgery (t3)*			
Sensory (NWC)	1.45 (2.29)	0.96 (2.46)	0.026*
Visual analogue scale	1.13 (1.36)	0.68 (1.63)	0.012*

* *P*<0.05.

).

Arm/shoulder pain was reported in only 36% of patients after SLNB in comparison to 68% after ALND at t2. While the number of patients with pain decreased significantly over time in the ALND group at t3 (Cochran's Q, *P*=0.008), no significant changes could be found in the SLNB group (*P*=0.08). Arm symptoms assessment by the EORTC QLQ-BR23 questionnaire showed no significant difference in both the groups at t3 (Table 3).

## DISCUSSION

The present study confirms previous observations suggesting that SLNB is associated with less arm/shoulder morbidity (Giuliano *et al*, 2000; Schrenk *et al*, 2000; Burak *et al*, 2002; Haid *et al*, 2002; Temple *et al*, 2002) than ALND. Evaluation and comparison of QOL outcomes in a short time follow-up in breast cancer patients undergoing ALND or SLNB after breast-conserving surgery provides additional observations:

(1) The type of axillary surgery does not seem to have an impact on global QOL, but may affect other QOL aspects as pain. (2) Body image and sexual functioning remain stable during the postsurgery follow-up in both types of axillary surgery. (3) The SLNB is associated with mild pain and mild sensory morbidity, significantly less than ALND, improving during the months following surgery. (4) Arm/shoulder abduction, flexion and horizontal adduction show significant impairment after ALND when compared with the preoperative range of motion.

The examination of postsurgery side effects after the different types of axillary surgery in our sample showed a significant difference in pain severity as well as in intensity of sensory morbidity of the affected arm after SLNB in comparison to ALND. Numbness was reported in 19.3% of the patients after ALND in contrast to 4% in the SLNB group, whereas ‘tugging’ was the most common complaint in both groups. The NWC shows that even after SLNB, a few patients experience substantial sensory complaints of the affected arm at t3. The properties of the McGill Pain Questionnaire in this matter are (1) exclusion of patients reporting breast pain, (2) specification of subjective pain intensity and (3) description of sensory qualities of pain by word descriptors as ‘numbing’, ‘tugging’, etc. Interestingly, evaluation of pain using the McGill Pain Questionnaire, the QLQ-C30 questionnaire and evaluation of ‘arm symptoms’ using the QLQ-BR23 questionnaire show some discrepancy. These results support the hypothesis, that current standard questionnaires do not cover all aspects of QOL (Janni *et al*, 2001). Several aspects of morbidity including pain, range of motion and sensory complaints of the affected arm have been reported to show significant difference in favour of SLNB (Giuliano *et al*, 2000; Schrenk *et al*, 2000; Burak *et al*, 2002; Haid *et al*, 2002; Temple *et al*, 2002). However, measuring instruments and scoring systems used in these studies differ widely.

In our study, patients' clinical and sociodemographic characteristics regarding age, tumour stage, adjuvant treatment were well balanced between the two groups. Using the EORTC QOL-C30 questionnaire, no significant difference could be detected in global QOL after ALND and SLNB at a short time follow-up. Interestingly, baseline assessment showed low levels of patients' global QOL in both groups increasing during follow-up. Statistically significant higher levels of global QOL are observed at t2 and t3 after SLNB, when compared with baseline levels. In contrast, impairment of global QOL at t2 after ALND clearly shows a difference in QOL improvement in favour of the SLNB group. We suggest that this is because patients in the SLNB group recover sooner than after ALND. However, patients having positive nodes in the ALND group reflect a more advanced disease. Randomised trials, as the ALMANAC trial, can possibly demonstrate the impact of axillary status on QOL. In addition to global QOL, assessment of emotional functioning shows low levels at baseline too, with no significant changes during follow-up in both groups. An explanation for low levels at baseline is that patients being informed about the breast cancer diagnosis before surgery induced psychological distress (Fallowfield *et al*, 1986; Ganz *et al*, 1992; Coscarelli Schag *et al*, 1993). In this study the Karnofsky performance status score was, for most patients, over 90 at baseline and showed no differences in patients' physical condition in both groups during follow-up. Using the EORTC QLQ-BR23 questionnaire comparison of body image and sexual functioning showed no difference between the two groups.

In the present study, we used a variety of validated measurement instruments to specify reliably patients' subjective experience of postoperative morbidity and QOL after SLNB in comparison to ALND. To our knowledge, this is one of the first reports to compare various aspects of QOL and arm/shoulder morbidity after different types of axillary surgery considering presurgery assessments. However, despite the analysis of many covariates with different measurement instruments a potential limitation of our study may be the small sample size.

In conclusion, the SLNB as a minimal invasive technique for axillary staging seems to be an alternative to ALND associated with a better postsurgery arm/shoulder mobility, with less pain and less sensory morbidity of the affected arm in a short-time follow-up. Severity of post-treatment side effects and QOL aspects should be considered when counselling breast cancer patients.
